# The net tax-benefit position and the role of self-interest in attitudes towards the welfare state

**DOI:** 10.23889/ijpds.v8i2.2343

**Published:** 2023-09-14

**Authors:** Solhee Han

**Affiliations:** 1 University of Oxford, Oxford, United Kingdom

This paper examines to what extent the actual net tax-benefit position (redistribution outcome) affects support towards the welfare state besides the typical indicator for self-interest, income. This paper then explores how own and partner’s socio-demographic factors affect support based on self-assessed social risks while ideology and beliefs mitigate self-interested judgements.

This paper integrates tax and benefit information from Luxembourg Income Study and attitudes data in European Social Survey using Statistical Matching. The technique integrates target variables that are distinctively observed in two sources using common variables which best explain the target variables in each data set. Using the linked data set, it adopts a multi-level model to explore institutional and individual factors. At the macro level, it investigates progressivity and sizes of taxes and transfers of nineteen European countries. Also, household arrangements including living with children and/or partner and demographics including age, gender, education, occupation, and own economic outlook are examined.

Using a more direct indicator for self-interest, the net tax-benefit position, expected results of the analysis are in three folds. First, those who pay more taxes and receive less benefits are less supportive of the welfare state despite the same income level. Second, those who are more likely to benefit from the welfare state are more likely to be supportive of the welfare state. For example, those who live with children may be more supportive due to higher likelihood to receive family benefits. Similarly, subjective assessment to benefit from the welfare state due to higher social risks such as unemployment or sickness leads to more positive attitudes. Third, those who are politically left and have less meritocratic values are likely to be more supportive.

Due to the data limitation, the impact of income-tax-benefit positions on welfare attitudes was not directly measured. This paper contributes to this discussion using the Statistical Matching and Multi-level modelling which explores interactions of actual net tax-benefit position, potential benefits based on social position/risks, and mitigating beliefs.

